# Diversity of Activities and Loneliness Among Heterosexual and LGBT Older Adults

**DOI:** 10.1093/geroni/igab046.133

**Published:** 2021-12-17

**Authors:** Jeongeun Lee, Joseph Svec

**Affiliations:** Iowa State University, Ames, Iowa, United States

## Abstract

The extant literature highlights the physiological and psychological benefits of active lifestyles among older adults, though there is a considerable gap in scholarship for sexual minority groups. Utilizing the Social Integration Model, we hypothesize that social activities enhance individual psychological well-being, but those effects differ by one’s social identities. Using a national AARP foundation survey of adults (45+), this study examines whether individuals’ activities predict loneliness and depressive symptoms of heterosexual (n=2905) and LGBTQ adults (n=318). We utilize an index of diverse activities, which includes, social technology use, meeting with friends, and volunteer activities. Multiple linear regression is used to study cross-sectional associations of loneliness and depressive symptoms on the diverse activity index. Results show that a wider array of activities correspond with higher psychological well-being and lower loneliness, and this association is higher for LGBTQ older adults. We discuss implications for counseling and wellness programming for LGBT older adults.

